# Estimation of Cyclic Stress–Strain Curves of Steels Based on Monotonic Properties Using Artificial Neural Networks

**DOI:** 10.3390/ma16145010

**Published:** 2023-07-15

**Authors:** Tea Marohnić, Robert Basan, Ela Marković

**Affiliations:** Faculty of Engineering, University of Rijeka, Vukovarska 58, 51000 Rijeka, Croatia; robert.basan@riteh.hr (R.B.); emarkovic@riteh.hr (E.M.)

**Keywords:** artificial neural networks, estimation, steels, cyclic stress–strain curves, cyclic stress–strain parameters

## Abstract

This paper introduces a novel method for estimating the cyclic stress–strain curves of steels based on their monotonic properties and plastic strain amplitudes, utilizing artificial neural networks (ANNs). ANNs were trained on a substantial number of experimental data for steels, collected from relevant literature, and divided into subgroups according to alloying elements content (unalloyed, low-alloy, and high-alloy steels). Only monotonic properties that were proven to be relevant for the estimation of points on the stress–strain curve were used. The performance of the developed ANNs was assessed using an independent set of data, and the results were compared to experimental values, values obtained by existing empirical estimation methods, and by previously developed ANNs. The results showed that the new approach which combines relevant monotonic properties and plastic strain amplitudes as inputs to ANNs for cyclic stress–strain curve estimation is better than the previously used approach where ANNs estimate the parameters of the Ramberg–Osgood material model separately. This shows that a more favorable approach to the estimation of cyclic stress–strain behavior would be to directly estimate corresponding material curves using monotonic properties. Additionally, this may also reduce inaccuracies resulting from simplified representations of the actual material behavior inherent in the material model.

## 1. Introduction

In various industries such as aerospace, automotive, and civil engineering, the reliability and safety of structures are of the highest importance. With the development of computer-aided engineering simulations, it is now possible to simulate the behavior and durability of engineering structures under real-world conditions. However, to obtain predictions on the behavior of structures under various loadings, a good understanding of the mechanical behavior of the materials used in these structures, which requires experimental determination, is essential. Monotonic testing of materials can provide valuable insight into material behavior, but since most structures in real-world applications experience cyclic loadings, knowledge of the materials’ behavior under cyclic loadings is also necessary.

Through cyclic experiments, parameters of the cyclic Ramberg–Osgood (R–O) equation [1], a widely used representation of the cyclic stress–strain response of the majority of metallic materials, can be determined:

(1) $$\frac{∆\varepsilon }{2}=\frac{∆\varepsilon_{e}}{2}+\frac{∆\varepsilon_{p}}{2}=\frac{∆\sigma }{2E}+{\left(\frac{∆\sigma }{2K\prime }\right)}^{\frac{1}{n\prime }}$$

where Δ*ε*, Δ*ε*_e_, and Δ*ε*_p_ are true total, elastic, and plastic strain ranges, respectively; Δ*σ* is the true stress range, *E* is the Young’s modulus, *K*′ is the cyclic strength coefficient, and *n*′ is the cyclic strain hardening exponent.

As opposed to monotonic tensile or compression tests, cyclic tests are more time-consuming, expensive, and not feasible for multiple candidate materials. Consequently, since the 1960′s, there is a constant and even growing interest in the development of estimation methods with which it would be possible to accurately predict fatigue parameters, and, somewhat later, also cyclic (R–O) parameters from easily obtainable monotonic parameters. Estimation methods that have been developed since range from empirical methods mostly based on the simple or multiple, linear or nonlinear regression models, to recent efforts focused on the development of more advanced, machine learning-based estimation models.

## 2. Overview and Analysis of Existing Approaches and Estimation Methods

### 2.1. Analytical and Empirical Methods

Zhang et al. [2] proposed a method for estimation of the cyclic parameters *K*′ and *n*′ of steels, aluminum, and titanium alloys by separating them into three groups depending on the value of a newly developed parameter called fracture ductility α. Using this method, it is possible to estimate cyclic parameters through the monotonic values of Ramberg–Osgood parameters, strength coefficient *K* and strain hardening exponent *n.* Basan et al. [3] developed a method for the correlation of experimentally determined cyclic parameters from the hardness of quenched and tempered low-alloy steel, 42CrMo4. A simple nonlinear relationship was proposed between *K*′ and Brinell hardness *HB* which showed a good correlation, whereas no such correlation could be found between *n*′ and *HB*. Lopez and Fatemi [4] performed an estimation of cyclic parameters from monotonic tensile data consisting of 123 steels of different chemical compositions and mechanical properties. To better estimate the cyclic strength coefficient *K*′, steels were grouped into three subgroups depending on the ratio of ultimate tensile strength *R*_m_ to yield strength *R*_e_. Expression for the estimation of *n*′ was performed on the whole dataset. In comparison to using all material data to find the relationship between monotonic tensile properties and *K*′, this grouping method increased the correlation between mentioned properties.

Li et al. [5] developed a method for estimating cyclic Ramberg–Osgood parameters *K*′ and *n*′ as well as cyclic yield strength *R*_e_′ from monotonic tensile properties. Expression for estimating the cyclic yield strength *R*_e_′ was originally developed in [6] and later modified, which resulted in a high coefficient of determination *R*^2^ of 96.1%. Expressions for estimation of cyclic strength coefficient *K*′ were developed on a dataset consisting of 121 steels with separation of steels in subgroups depending on the ratio of tensile strength to yield strength *R*_m_/*R*_e_. Using the values of *K*′ obtained from these expressions for estimating *K*′, it was possible to determine cyclic strain hardening exponent *n*′. 

In a more recent study, Zonfrillo [7] proposed a new method for estimating the cyclic strength coefficient *K*′ and cyclic strain hardening exponent *n*′ exclusively from properties obtained from monotonic tensile tests and their nonlinear combinations. The study focused on three types of materials: iron alloys (primarily steel), titanium alloys, and aluminum alloys. Expressions were developed separately for each group. In this publication, a novel grouping method to improve the correlation of *n*′ for each material type was introduced using true fracture ductility *ε*_f_ and reduction of area at fracture *RA*. The results showed that Zonfrillo’s method demonstrates higher accuracy in predicting both *K*′ and *n*′ when compared with other expressions from the literature.

These analytical estimation methods are still not abandoned due to their practicality, simplicity, and quickly obtainable results, which can be seen in a recent paper by Derrick and Fatemi [8], where correlations of fatigue strength of additively manufactured metals with hardness and defect size were established.

There are several challenges in estimating the cyclic Ramberg–Osgood parameters. Empirical methods that rely only on monotonic parameters such as *R*_m_, *R*_e_, or *HB* often provide insufficient results [7], which supports the claim that no useful correlation has been found between *n*′ and the hardness value *HB* [3]. Assigning average or median values to these parameters due to the difficulty in determining functional relationships between monotonic and cyclic parameters is inadequate, as different groups of metallic materials have significant differences in their cyclic behavior and corresponding parameters, as shown by Marohnić et al. [9].

### 2.2. Machine Learning-Based Approaches and Estimation Methods

Following the discussion on empirical methods for the estimation of cyclic parameters and presented difficulties, recent trends in the application of machine learning in material science in general and also in the development of more advanced estimation methods have shown some promising results. Machine learning methods, more precisely, Artificial Neural Networks (ANNs), are particularly gaining popularity and increased application due to their ability to identify complex relationships between inputs (predictor variables) and outputs (dependent variables), providing a more accurate and reliable estimation of material parameters applied to various fields of material science. Kalla et al. [10] developed an ANN model for the estimation of machining process outcomes of composite materials that showed significant improvements in the predictive power as opposed to multiple regression methods. Asiltürk and Çunkaş [11] employed ANN to determine the surface roughness of AISI 1040 steel after the turning process and reported a coefficient of determination *R*^2^ of 98.9%. Recently, Zhang et al. [12] developed a model for estimating the high cycle fatigue life of laser powder bed fusion stainless steel 316L using the neuro-fuzzy-based machine learning technique. Similarly, Bao et al. [13] adopted a machine learning method to explore the influence of defect parameters on the fatigue life of selective laser-melted Ti-6Al-4 V alloy, achieving high coefficients of determination. Most recently, Jia et al. [14] examined the fatigue behavior of titanium alloys fabricated by laser powder bed fusion, particularly in very-high-cycle fatigue regimes. They developed a deep-belief neural network for predicting the fatigue life of such alloys. These studies demonstrate the increasing use of machine learning methods in predicting fatigue material behavior, especially in the emerging field of additive materials. Furthermore, it is worth noting the recent study conducted by Mosleh et al. [15], where they developed an ANN model for predicting the mechanical properties of anti-friction aluminum-based alloys. The mechanical properties considered were ultimate tensile strength, elongation to failure, and hardness, with the chemical compositions of the alloys serving as inputs. The model exhibited high quality compared with unseen experimental data.

The utilization of ANNs has also been demonstrated in several research papers regarding the estimation of cyclic and fatigue parameters specifically related to steel based on its monotonic parameters. For example, Genel [16] developed four separate artificial neural networks for predicting each of the individual Basquin–Coffin–Manson (B–C–M) parameters. The material dataset included 73 steels and the reported accuracy was around 99% for fatigue strength and ductility coefficients. Troshchenko et al. [17] also employed four ANNs to estimate B–C–M parameters and their approach resulted in lower maximum relative errors compared to conventional methods. Networks were trained on data consisting of 140 various steels including carbon, low- and high-alloy steel. In a more recent study, Soyer et al. [18] predicted the fatigue parameters of high-alloy steel at low cycles using one artificial neural network with four outputs and achieved an accuracy of 99% on test data. Their whole dataset included 38 high-strength steels from which only 10% was used for testing with five inputs representing common monotonic tensile parameters and four outputs being B–C–M parameters.

Apart from predicting Basquin–Coffin–Manson parameters, several authors have demonstrated the successful development of ANN models for the estimation of Ramberg–Osgood parameters. Ghajar et al. [19] constructed two separate neural networks to predict cyclic Ramberg–Osgood parameters *K*′ and *n*′ from monotonic tensile properties. Their dataset included 48 various steels for the first neural network used for predicting *K*′ and 82 steels for the second neural network used for predicting *n*′ of which around 25% was used for testing the networks. The reported testing accuracy for predicting *K*′ and *n*′ was 95.3% and 86.5%, respectively. Tomasella et al. [20] developed an artificial neural network for simulating virtual experimental tests instead of directly predicting cyclic material properties *K*′ and *n*′. From the selected strain amplitudes *ε*_a_, the number of cycles to failure *N*_f_ and stress amplitude *σ*_a_ was calculated, which was then used as an output of ANN. They reported better accuracy of their approach when compared to the chosen empirical method. More recently, Marohnić [21] developed a new approach to the estimation of cyclic and fatigue parameters of unalloyed, low-alloyed, and high-alloyed steels that includes the identification of monotonic properties relevant for the estimation of each particular cyclic and fatigue parameter and each steel subgroup and incorporating that knowledge to modeling of artificial neural networks for the considered problem. Separate ANNs were developed for each cyclic Ramberg–Osgood parameter as well as strain-life Basquin–Coffin–Manson parameter. For the testing dataset, ANNs proved to be more successful than empirical methods for estimating most of the cyclic and fatigue parameters.

From the literature review and analysis of existing approaches and methods, two main drawbacks can be identified. First, most of the existing studies developed models on the datasets consisting of the variety of steel without considering differences in individual subgroups of steel. To counter this, in the presented study, the estimations were performed separately for steels grouped according to alloying element content since in [9,21] it was shown that the cyclic stress–strain behavior of these groups is statistically significantly different.

Secondly, most of the previous approaches for estimating cyclic or fatigue material behavior included separate estimation of parameters of various models, particularly the cyclic Ramberg–Osgood parameters, and subsequent calculation of stress amplitudes Δ*σ*/2 using those parameters [21,22]. Estimating model parameters inherently introduces errors, as these models are not perfect representations of the actual material behavior. A more favorable approach would be to directly estimate material response in the form of values of stress amplitudes correspondent to a selected set of strain amplitudes using monotonic data. By doing so, points on the cyclic stress–strain curve could be directly estimated using experimental data points. Consequently, the parameters of any model could be determined based on the cyclic stress–strain curve obtained through this approach.

Additionally, the aforementioned approaches do not include detailed statistical analysis of monotonic parameters relevant to the estimation of cyclic and/or fatigue parameters and material behavior.

Such direct prediction of stress–strain curves using a neural network approach has already proven successful in various fields. One such example is the work of Gangi Setti and Rao [23], who developed an ANN model for predicting the monotonic stress–strain curve of a titanium alloy. In their study, the input features consisted of strain values and different microstructures of the titanium alloy, specifically represented as the volume fraction of the α phase and the stress values served as the target values for the ANN. Similarly, Yang et al. [24] used a machine learning approach and combined principal component analysis with a convolutional neural network (CNN) to predict the stress–strain curves of binary composites. The input feature to CNN included a binary matrix representing the composite design, generated through finite element analysis, whereas the target variable was a stress vector. Lavech du Bos et al. [25] developed a methodology for the modeling of stress–strain curves of one fully reversed loading cycle using the ANN approach, thus avoiding the determination of model parameters necessary for defining a constitutive model. The model was trained on data generated using a simulation algorithm.

In the performed literature review, no references were found dealing primarily with the estimation of parameters of more advanced constitutive material models from monotonic properties and related material information. A study indicating such a possibility was performed by Basan et al. [26], in which parameters of a rate-independent Chaboche model were calculated based on cyclic stress–strain curves which were determined using a simple Ramberg–Osgood model. Satisfactory approximations were obtained for a set of differently heat-treated low-alloy steel, 42CrMo4.

The main aim of this paper was to explore the possibility of developing and utilizing artificial neural networks to estimate materials’ response, i.e., stress amplitudes Δ*σ*/2, using relevant monotonic properties and selected plastic strain amplitudes Δ*ε*_p_/2 as inputs to artificial neural networks. Combining the findings from [26] and the approach to the estimation of cyclic stress–strain curves and Ramberg–Osgood parameters from monotonic properties of materials developed by Marohnić [21,22] might also result in the possibility of reasonable estimations of parameters of other, more advanced constitutive models describing materials’ stabilized stress–strain responses using machine learning, specifically ANNs. Additionally, the direct estimation of materials’ responses, instead of estimating their parameters, in this case, the Ramberg–Osgood model for cyclic stress–strain behavior, can enable better estimations. The results will be compared to those obtained using empirical methods available in the literature and using previously developed ANNs [21,22].

## 3. Materials and Methods

### 3.1. General Methodology

As proposed earlier, estimations will be performed separately for steels grouped according to alloying element content since it was shown in [9,21] that the cyclic stress–strain behavior of these groups statistically significantly differed.

In the framework of this study, for estimation of cyclic stress–strain behavior, i.e., curves, monotonic properties are used, which were proved to be statistically significant for the estimation of cyclic yield stress *R*_e_′ for each steel group (Table 1) as published in an earlier study [27]. Since it was proved there that *R*_e_′, as a point on the cyclic stress–strain curve, can be successfully estimated using monotonic properties, it is assumed that by adding plastic strain amplitudes Δ*ε*_p_/2 to monotonic properties already proven as statistically significant for estimation of *R*_e_′, the whole cyclic stress–strain curve can be successfully estimated. The motivation for determination and using only significant, i.e., relevant monotonic properties for estimation of cyclic stress–strain parameters and curves is obtaining a favorable ratio between the number of input variables and the number of available datasets for ANN training, as explained in more detail in Section 3.3. 

Details on the forward stepwise regression procedure used for the identification of relevant monotonic properties for estimation of cyclic yield stress *R*_e_′ are provided in [21,27].

**Figure 1:**
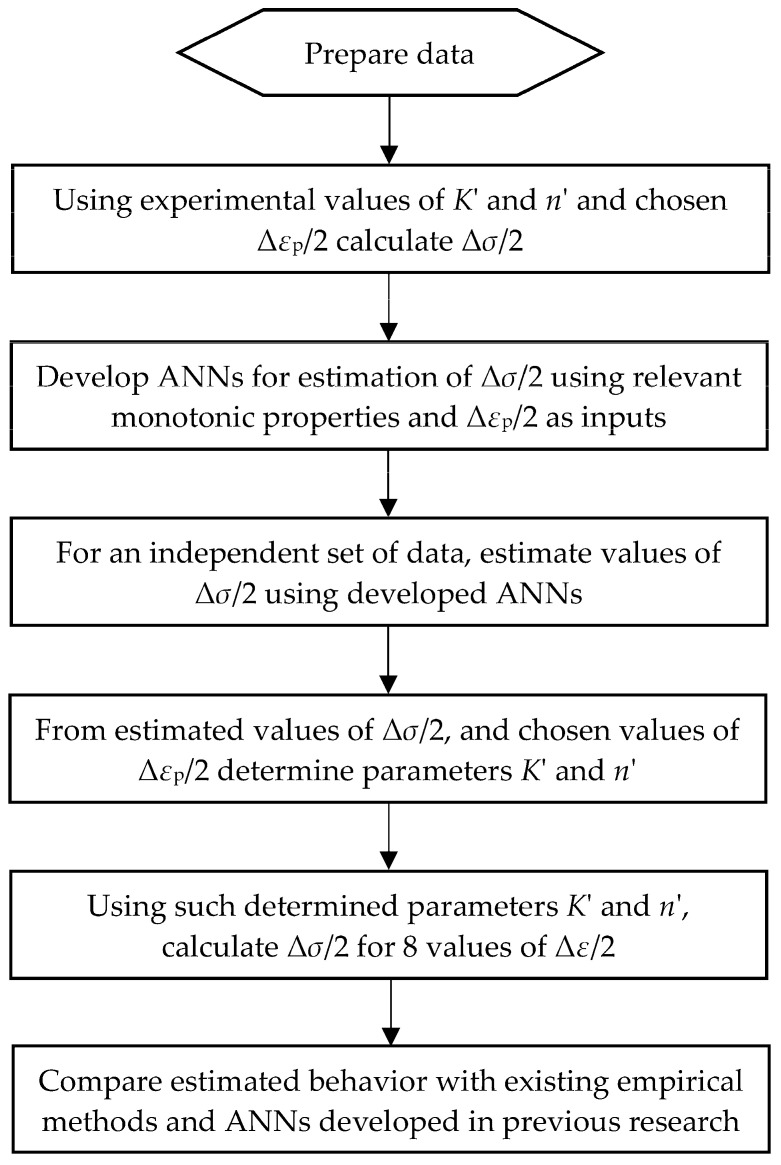
Flow chart of the cyclic stress–strain curves estimation using ANNs.

The procedure of estimation of cyclic stress–strain behavior is given in the flow chart in Figure 1. Even though most steps are easily understandable, the term “chosen values of Δ*ε*_p_/2” must be addressed. The investigation and results presented here are the first steps towards the development of the method for estimation of materials’ cyclic stress–strain behavior using experimental data points instead of (cyclic) stress–strain curve points calculated using a certain model and the corresponding material parameters. As a result, the parameters of any appropriate material model could then be determined. This investigation, however, is based on the calculation of those data points (Δ*σ*/2–Δ*ε*_p_/2) determined using the cyclic Ramberg–Osgood material behavior model. Since the plastic part of the cyclic stress–strain curve (Δ*σ*/2–Δ*ε*_p_/2) calculated using the R–O model is a straight line in the double logarithmic diagram, theoretically only two points—any two points on the curve—are needed for the determination of the line. However, since the aim of this research is to set foundations for comprehensive estimation of cyclic stress–strain curves using experimental data points, i.e., actual plastic strain amplitudes and monotonic properties as independent variables (inputs), in this investigation, two variations in the number of data points for each material were used:

1. Δ*σ*/2 for Δ*ε*_p_/2 = 0.2% and 2%;
2. Δ*σ*/2 for Δ*ε*_p_/2 = 0.2%; 0.5%, 1%, 1.5% and 2%.

The second option with multiple (5) values of plastic strain amplitudes serves to evaluate the performance of ANNs for a larger number of data points.

### 3.2. Materials Data

Statistical analysis for the determination of the relevance of particular monotonic properties for the estimation of cyclic stress–strain parameters [21,27] was performed on data for three representative groups of steels: unalloyed (UA), low-alloy (LA), and high-alloy (HA) steels collected from the relevant literature. For the analysis, only data from strain-controlled, fully reversed (*R* = −1) axial cyclic tests conducted in the air at room temperature, performed on at least four different strain amplitudes with a range of total strain amplitudes greater than 0.4%, were taken into account. There were a total of 34 UA, 47 LA, and 35 HA steels that could be analyzed. Steels were subjected to different processing and heat treatment so datasets covered a wide variety of conditions.

Detailed material data used for performing the forward selection of relevant monotonic properties for the estimation of each cyclic parameter of each group of steels were collected from [28,29] through [30]. Summarized tables can be found in [21,27]. In Table 2, minimum/maximum/mean values of the key monotonic properties and cyclic parameters can be found, along with alloying element content range (wt.%) for unalloyed, low-alloy, and high-alloy steels, respectively.

Since the number of datasets used for statistical analysis is small in the context of ANNs, so additional data were collected for the development and evaluation of ANNs. Data were acquired [28,30,31,32,33,34,35,36,37,38,39,40,41,42] using the same criteria and respecting the same distribution as those used for statistical analysis [21,27], ensuring that the findings reached are applicable to the new data as well. A portion of the newly acquired datasets was used for an independent evaluation of the ANN methodology (comparison with experimental results, estimations obtained using selected empirical methods and previously developed ANNs), but not for training ANNs. In total, 59 experimental datasets for unalloyed steels, 70 for low-alloyed steels, and 35 for high-alloyed steels were used for training ANNs for the estimation of stress amplitudes Δ*σ*/2. A total of 52 datasets were available for unbiased evaluation of ANNs, including 17 unalloyed, 25 low-alloy, and 10 high-alloy steels.

The datasets used for the development and evaluation of ANN results in this research are the same as in [21,22] in order to obtain comparable results. Detailed materials data and a list of data sources used are provided in [21,22].

### 3.3. ANNs for Estimation of Cyclic Stress–Strain Behavior

Although regression models are quite accurate approximations for most regression problems, their capacity to discover more complicated correlations between input variables (predictors) and output (dependent) variables is restricted. Artificial neural networks, in contrast to regression models, are nonlinear, adaptable computational models employed in a variety of fields. They are frequently used for function approximation, which is the detection of an unknown relationship between input and output variables, among its many applications. ANNs can therefore be utilized to predict cyclic stress–strain behavior.

**Figure 2:**
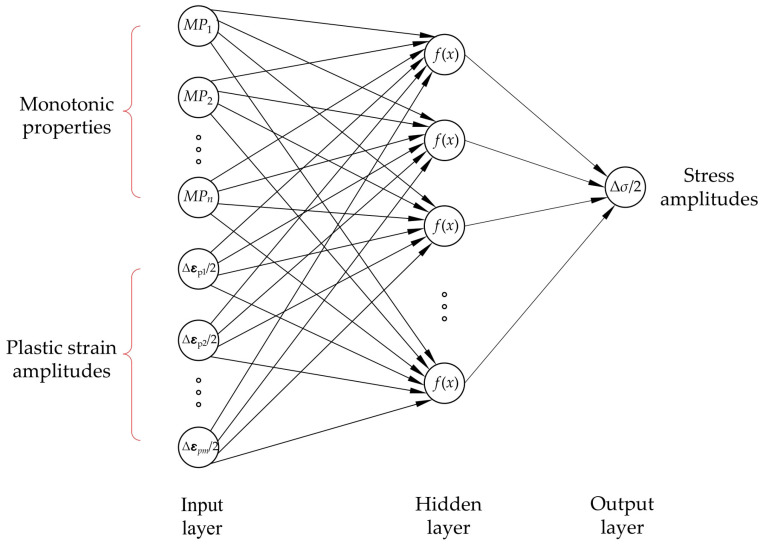
A fully connected multilayer perceptron with one hidden layer was employed in this study.

ANNs are based on biological neural networks and consist of three layers: input layer (one neuron for each input variable), hidden layers (one or more neurons in one or more hidden layers), and output layer (one neuron for each output variable). Figure 2 shows a schematic representation of one-layered ANNs employed in this study.

Six two-layer multilayer perceptrons (MLP) with a hyperbolic tangent transfer function in the hidden layer and a linear transfer function in the output layer were developed in order to estimate the cyclic stress–strain behavior of UA, LA, and HA steels. The training parameters of ANN models are given in Table 3.

The main objective of ANN development is to adjust weights that connect neurons so that output variables (estimated values of Δ*σ*/2) are close to target values (experimental values of Δ*σ*/2). This is met when the error function, in this instance mean square error (*MSE*), has reached its minimum.

Forward selection defined the input variables (monotonic properties), as explained previously and given in Table 1, along with the chosen values of Δ*ε*_p_/2 (two or five different values) for which the corresponding values of Δ*σ*/2 were calculated and used as the multilayer perceptron target. The methodology of ANN development relies on that given in [21,22].

The maximum number of neurons in hidden layer *H* is defined by the number of training samples available, *N*_train_, the number of input variables *I,* and the number of target variables *O*:

(2) $$H\leq \frac{O\cdot \left(N_{train}-1\right)}{I+O+1}$$

The number of neurons in the hidden layer was determined by combining the growth method (starting from one neuron in the hidden layer) with a method for improving generalization—early stopping in order to prevent overlearning (in extreme cases, overlearning leads to memorizing training data), i.e., improve generalization (performance of the network on new, “unseen” data). For learning (i.e., minimizing error function), Levenberg–Marquadt algorithm with early stopping is used, being the most effective as shown in previous investigations.

A common caveat of ANN modeling is overlearning, which in some extreme cases can lead to memorizing training data so that ANNs perform poorly on new, unseen data, i.e., are unable to generalize well. Thus, in this study, a growth method was used for determining the number of neurons in the hidden layer (starting from one neuron) and combined with a learning algorithm with early stopping to improve the generalization of ANNs. Levenberg–Marquadt algorithm with early stopping is used for learning, i.e., minimizing error function, which is the most effective, as shown in previous investigations. Because the number of accessible data for creating ANNs is relatively small in terms of ANN modeling, but quite large in contrast to existing methods of cyclic parameter/behavior estimation, *k*-fold cross-validation was utilized, with *k* set to ten folds. *k*-fold cross-validation is an effective approach to make the most of the data available for ANN development. A sample is partitioned into ten subsamples at random, and in each training, one subsample is used for ANN validation (or testing). Finally, k networks are modeled, and an average of all of them is used for evaluation. Ensembles of networks, also known as committees of networks, combine the findings of two or more ANNs to produce a better level of generalization on unseen data. For each ANN architecture (number of neurons in hidden layer), 10 ensembles were trained with 10 different initial values of weights per one architecture (hidden layer size *H*) to reduce the possibility of the error function of the selected network converging to local instead of global minimum. In total, 370 ensembles (3700 networks) were trained for estimations based on two values of Δ*ε*_p_/2, and 950 ensembles (9500 networks) for estimations based on five values of Δ*ε*_p_/2 using MATLAB R2022b [43].

The best ensembles were chosen from all architectures based on the value of the error function (in this case the mean square error) and evaluated on an independent set of data that had not been utilized for ANN training. Table 4 shows the root mean square error *RMSE*_mean,test_ and coefficient of correlation *r*_test_ between targets and outputs (i.e., experimental and estimated values) for each considered ensemble.

## 4. Results

After ANNs were developed, selected networks had to be tested on unseen data to obtain their predictive accuracy. Designation of ANN approaches that differ in inputs and target is as follows: estimation using two data points (Δ*ε*_p_/2 = 0.2% and 2%) on the plastic part of the cyclic stress–strain curve—ANN-CSSC-2, estimation using five data points (Δ*ε*_p_/2 = 0.2%; 0.5%, 1%, 1.5%, and 2%) on the plastic part of the cyclic stress–strain curve—ANN-CSSC-5.

**Figure 3:**
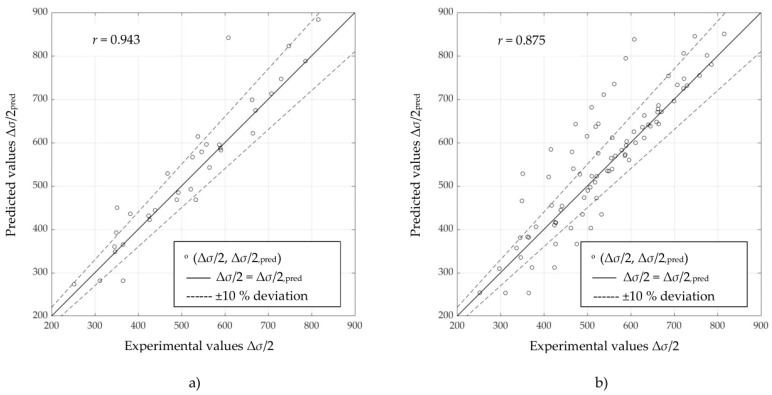
Scatter diagrams of stress amplitudes Δ*σ*/2 obtained by selected artificial neural networks vs. experimental counterparts for (**a**) ANN-CSSC-2 approach and (**b**) ANN-CSSC-5 approach, on “unseen” data for unalloyed steels.

**Figure 4:**
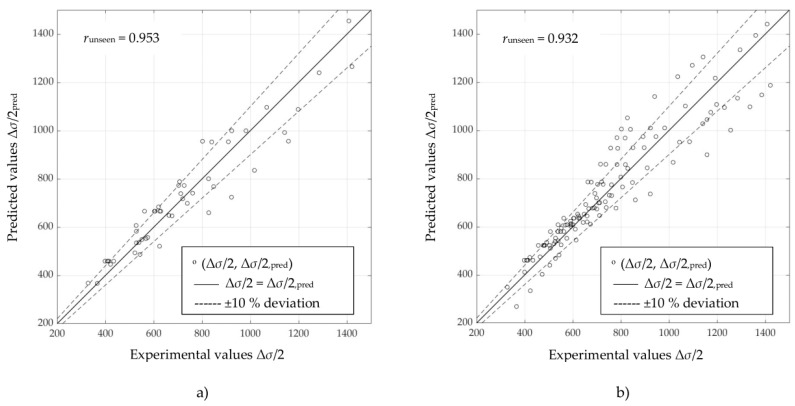
Scatter diagrams of stress amplitudes Δ*σ*/2 obtained by selected artificial neural networks vs. experimental counterparts for (**a**) the ANN-CSSC-2 approach and (**b**) the ANN-CSSC-5 approach, on “unseen” data for low-alloy steels.

**Figure 5:**
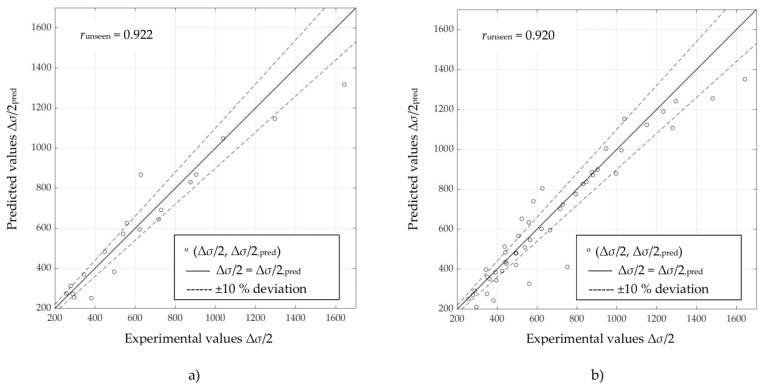
Scatter diagrams of stress amplitudes Δ*σ*/2 obtained by selected artificial neural networks vs. experimental counterparts for (**a**) ANN-CSSC-2 approach and (**b**) ANN-CSSC-5 approach, on “unseen” data for high-alloy steels.

Scatter diagrams in Figure 3, Figure 4 and Figure 5 represent information on the relation between experimental and predicted values of stress amplitude values Δ*σ*/2, obtained directly using selected ANNs (Table 4) on “unseen” data for unalloyed, low-alloy and high-alloy steels, respectively. Figure 3a, Figure 4a, and Figure 5a are obtained using ANN-CSSC-2, while Figure 3b, Figure 4b, and Figure 5b are obtained using ANN-CSSC-5 approach.

It can be seen that for ANN-CSSC-2 consistently no significant deviation of data to either side of the regression line (*r* = 1) can be observed, while for ANN-CSSC-5 the scatter is more pronounced, especially for unalloyed and low-alloy steels (Figure 3b and Figure 4b respectively). Using ANN-CSSC-5 for unalloyed steels results in a significant number of Δ*σ*/2 that are either underestimated or overestimated, especially within the region of experimental values of Δ*σ*/2 between 350 MPa and 600 MPa. For low-alloy steels, ANN-CSSC-5 results in a scatter that is more pronounced for experimental values of Δ*σ*/2 higher than approximately 700 MPa.

## 5. Performance Evaluation of ANNs and Comparison to Existing Approaches

To further evaluate the predictive accuracy of artificial neural networks for the estimation of stress amplitudes Δ*σ*/2 and to compare performance to approaches available in the literature, results were compared to those obtained by existing empirical methods ([2,4,5]) for estimation of *K*′ and *n*′ and previously developed ANNs, i.e., ANN-param, that directly estimate RO parameters [21,22]. Details of selected methods are given in [9,21]. It should be noted that only three datasets were available for evaluation of the Zhang 1 method for high-alloy steels.

In order to validate networks, stress amplitudes were first estimated using selected ANNs (Table 4), and then parameters *K*′ and *n*′ were determined from estimated values of Δ*σ*/2, and chosen values of Δ*ε*_p_/2, which were then used to calculate values of stress amplitudes Δ*σ*/2 for eight different total strain amplitudes ∆*ε*/2: 0.25%, 0.3%, 0.35%, 0.4%, 0.45%, 0.5%, 0.9%, and 1.5%. For obtained values, deviations up to ±5, ±10, ±15, and ±20% from Δ*σ*/2 calculated using experimental values of *K*′ and *n*′ were determined.

**Figure 6:**
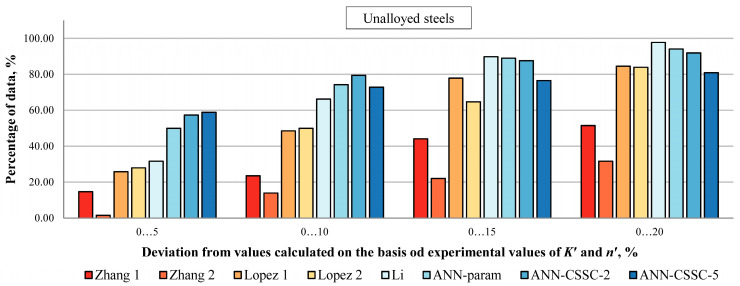
Percentage of Δ*σ*/2 values estimated by selected empirical methods and various approaches to ANNs that deviate up to 5, 10, 15, and 20% from values calculated on experiment-based values of cyclic stress–strain parameters for unalloyed steels [2,4,5,22].

**Figure 7:**
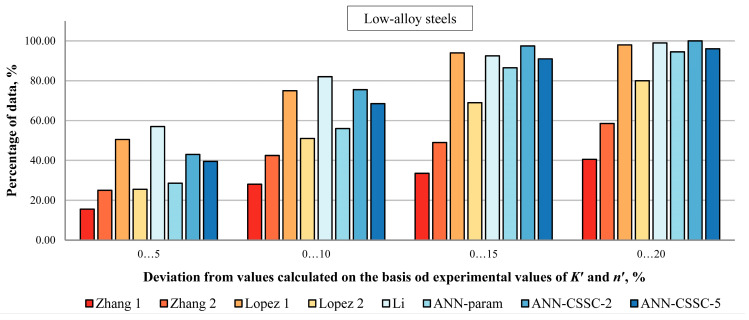
Percentage of Δ*σ*/2 values estimated by selected empirical methods and various approaches to ANNs that deviate up to 5, 10, 15, and 20% from values calculated on experiment-based values of cyclic stress–strain parameters for low-alloy steels [2,4,5,22].

**Figure 8:**
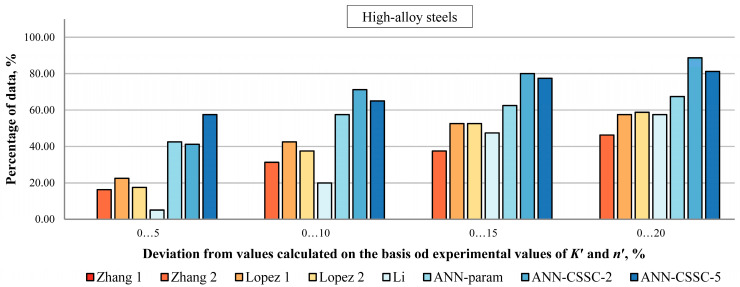
Percentage of Δ*σ*/2 values estimated by selected empirical methods and various approaches to ANNs that deviate up to 5, 10, 15, and 20% from values calculated on experiment-based values of cyclic stress–strain parameters for high-alloy steels [2,4,5,22].

In Figure 6, Figure 7 and Figure 8, the percentage of Δ*σ*/2 values estimated by selected empirical methods and different models of ANNs that deviate up to ±5, ±10, ±15, and ±20% from values calculated on experiment-based values of cyclic stress–strain parameters is given for unalloyed, low-alloy and high-alloy steels. 

For UA steels (Figure 6), ANNs provide better estimations in deviations up to ±10%, with the approach of direct estimation of stress amplitudes Δ*σ*/2 (ANN-CSSC-2 and ANN-CSSC-5) being somewhat more successful than the approach that separately estimates cyclic stress–strain parameters (ANN-param), around 58% versus 50%. From those two variations of direct estimation of stress amplitudes Δ*σ*/2, ANN-CSSC-2 is somewhat more successful than ANN-CSSC-5 when comparing deviations up to 20% (92% vs. 81%), and is comparable to ANN-param (94%).

For LA steels (Figure 7), direct estimation of stress amplitudes Δ*σ*/2 is significantly better than the ANN-param estimation approach in deviations up to ±5% (ANN-param 29%, ANN-CSSC-2 43%, ANN-CSSC-5 40%), up to ±10% (ANN-param 56%, ANN-CSSC-2 76%, ANN-CSSC-5 69%), and up to ±15% (ANN-param 87%, ANN-CSSC-2 98%, ANN-CSSC-5 91%). However, ANN-CSSC-2 and ANN-CSSC-5 are comparable to best empirical methods (Li) only in deviations up to ±20%, which is around 95–100% of data falling into that deviation range.

The most pronounced improvement is observed for HA steels (Figure 8). It was already proven with the ANN-param approach for parameter estimation that the estimated behavior is better than empirical methods. Here, ANN-CSSC-5 results in 58% of data deviating up to ±5% from experimental-based counterparts, compared to 23 and 43% obtained from Lopez 1 and ANN-param, respectively. For larger deviations, up to ±20%, ANN-CSSC-2 results in 89% of data falling within that range, compared to around 58% and 68% obtained by the best empirical method (Lopez 1, 2 and Li) and ANN-param respectively. 

Two additional statistical indicators of the model’s performance on “unseen” data are provided in Table 5 for all methods and approaches evaluated, for unalloyed, low-alloy, and high-alloy steels—mean absolute percentage error, *MAPE*, and root mean square error, *RMSE*, as in [21,22,44,45,46].

*MAPE* value is commonly interpreted as a prediction (forecasting) goodness indicator, with values under 10% indicating highly accurate forecasting. Sometimes it is considered to show the generalization capability of the model. It is calculated using Equation (3):

(3) $$MAPE=100\frac{1}{n}\sum_{i=1}^{n}\left|\left.\frac{x_{i\exp}-x_{i\mathrm{est}}}{x_{i\exp}}\right|\right.$$

where *x_i_*_exp_ are experimental values and *x_i_*_est_ are estimated values of Δ*σ*/2.

However, the determination of the generalization capability of the model should not be based solely on one indicator; thus, *RMSE* is calculated using Equation (4):

(4) $$RMSE=\sqrt{\frac{1}{n}\sum_{i=1}^{n}{\left(x_{i\mathrm{est}}-x_{i\exp}\right)}^{2}}$$

*RMSE* represents the standard deviation of estimation errors, i.e., the average difference between values that are estimated by a model, and experimental values.

The lowest values of *MAPE* and *RMSE* are underlined in Table 5 for each steel group, indicating models with the best generalization capability. For unalloyed steels, the lowest values of both *MAPE* and *RMSE* were observed for the ANN-CSSC-2 approach, closely followed by ANN-param [22]. For low-alloy steels, the lowest *MAPE* value is observed for the method by Li et al. [5] (Li 1), closely followed by Lopez 1 [4] and ANN-CSSC-2. However, ANN-CSSC-2 shows lower value of *RMSE* than both of these methods. For high-alloy steels, ANN-CSSC-2 and ANN-CSSC-5 showed significantly better results, i.e., the lowest values of both *MAPE* and *RMSE* compared to all other methods, with ANN-CSSC-5 being slightly better than ANN-CSSC-2.

## 6. Discussion and Conclusions

Figure 6, Figure 7 and Figure 8 show that the approach to the estimation of cyclic stress–strain curves proposed in this paper, as a preliminary investigation of direct estimation of cyclic stress–strain curves from monotonic properties and experimental data points, and a foundation for estimation of parameters of selected advanced constitutive models, provides comparable results as best empirical methods and previously developed ANN approach where cyclic stress–strain parameters are estimated separately.

**Figure 9:**
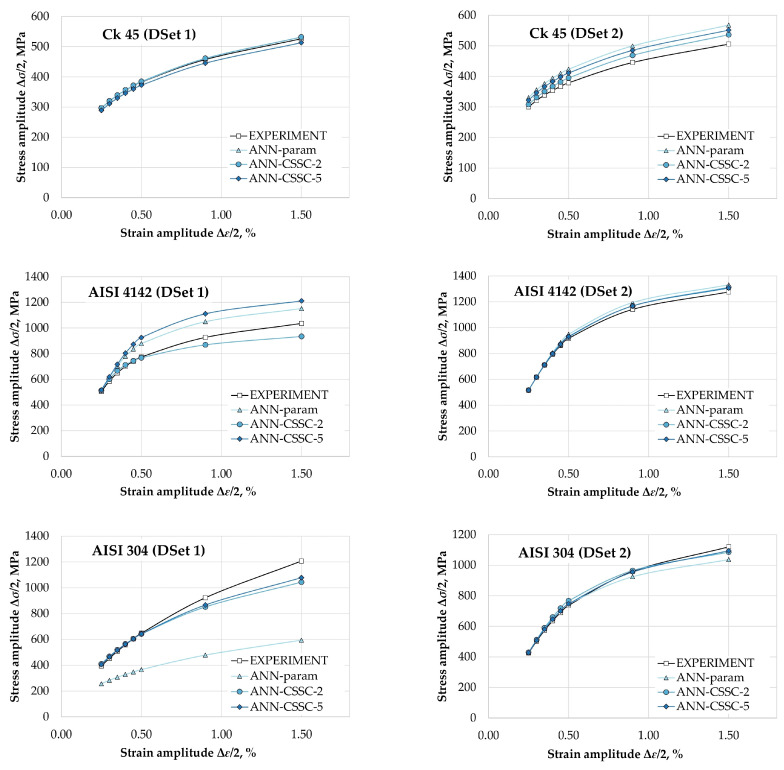
Experimental and points on cyclic stress–strain curves for DIN Ck 45, AISI 4142, and AISI 304 steel datasets using ANN-param [22], ANN-CSSC-2, and ANN-CSSC-5 approaches.

To get more insight into the performance of the proposed approach where monotonic properties and data points (Δ*σ*/2–Δ*ε*/2) were used to train an artificial neural network, in Figure 9, estimated points of cyclic stress–strain curves for DIN Ck 45, AISI 4142 and AISI 304 (2 datasets each) are given in comparison to their experimental counterparts for the three artificial neural network approaches: ANN-param [22], ANN-CSSC-2, and ANN-CSSC-5.

It can be seen that for Ck 45 (dataset 1), the results for ANN-CSSC-2 and ANN-CSSC-5 are comparable to the ANN-param approach, where monotonic properties are used to estimate *K*′ and *n*′ separately. Data points obtained by ANN-CSSC-2 are almost overlapping those obtained by ANN-param. For Ck 45 (dataset 2), it can be seen that all three approaches resulted in good, but slightly non-conservative estimations, with ANN-CSSC-2 being the closest to experimental data points. For AISI 4142 (dataset 2) and AISI 304 (dataset 2), the results were somewhat similar as for Ck 45 (dataset 1), although estimated data points deviated more from experimental curves for the region of Δ*ε*/2 > 0.4%. For these datasets, results obtained by ANN-CSSC-2 and ANN-CSSC-5 are better than using ANN-param, although AISI 4142 (dataset 2) were still somewhat nonconservative. Results for DIN Ck 45 (dataset 2) and AISI 4142 (dataset 1) showed significant differences between the three approaches. In both of these cases, estimations using ANN-CSSC-2 are better than the other two (especially ANN). For AISI 4142 (dataset 1), ANN-CSSC-2 provides conservative estimates, while ANN-param and ANN-CSSC-5 are nonconservative.

The most pronounced difference is seen for AISI 304 (dataset 1) where the ANN-param approach significantly underestimates the stress amplitudes. For the same dataset, data points obtained by ANN-CSSC-2 and ANN-CSSC-5 are almost overlapping within the region up until Δ*ε*/2 = 0.5%, while the portion above Δ*ε*/2 = 0.5% is much closer to experimental data than for ANN, and still in a conservative region.

When comparing ANN-CSSC-2 and ANN-CSSC-5 it is seen that the ANN-CSSC-2 approach, where only two points on the plastic portion of cyclic stress–strain curves (Δ*σ*/2 for Δ*ε*_p_/2 = 0.2% and 2%) are used for artificial neural network training, is, in general, better than ANN-CSSC-5, where five points on the plastic portion of cyclic stress–strain curves (Δ*σ*/2 for Δ*ε*_p_/2 = 0.2%; 0.5%, 1%, 1.5%, and 2%.) are used. This is probably due to some noise caused by more data points (Δ*σ*/2–Δ*ε*_p_/2) per one combination of monotonic properties used as inputs for artificial neural network training. One important thing to address is that the smoothness of the curves obtained by both ANN-CSSC-2 and ANN-CSSC-5 showed that overfitting, as a common risk accompanying ANN modeling, is adequately addressed.

The obtained results and performance evaluation show potential for the estimation of cyclic stress–strain curves and parameters of other constitutive models using monotonic properties and experimental data points in an artificial neural network approach. However, further investigations must be made regarding the relevance of monotonic properties, the number of data points used, the ANN algorithm, different ML algorithms, etc.

## Figures and Tables

**Table 1:** Overview of monotonic properties that proved to be relevant for estimation of cyclic yield stress *R*_e_′ of unalloyed, low-alloy, and high-alloy steels.

Steel Group	Independent Variables (Monotonic Properties)									
	Modulus of Elasticity *E*	Yield Stress *R*_e_ or *R*_p0.2_	Ultimate Strength *R*_m_	Ultimate Strength to Yield Stress Ratio *R*_m_/*R*_e_	Ultimate Strength to Modulus of Elasticity Ratio *R*_m_/*E*	Reduction of are at Fracture *RA*	Strength Coefficient *K*	Strain Hardening exponent *n*	True Fracture Stress *σ*_f_	Modulus of Elasticity *E*
UA		+	+			+		+		UA
LA			+	+			+			LA
HA		+	+		+	+	N/A	N/A	N/A	HA

**Table 2:** Maximum, minimum, and mean values of monotonic properties and cyclic parameters of datasets used for forward selection [21,27] and alloying element content range (wt.%) for unalloyed, low-alloy, and high-alloy steels [28,30,31].

Steel Subgroup	Value	Monotonic Properties											Cyclic Parameters		
		*HB* (HB)	*E* (MPa)	*R*_e_ or *R*_p0.2_ (MPa)	*R*_m_ (MPa)	*R*_m_/*R*_e_ (-)	*R*_m_/*E* (10^−3^)	*RA* (%)	*K* (MPa)	*n* (-)	*σ*_f_ (MPa)	*ε*_f_ (-)	*R*’_p0.2_ (MPa)	*K*′ (MPa)	*n*′ (-)
UA	Min	130	190,000	207	359	1.159	1.710	0	330	0.015	653	0.000	239	813	0.085
	Max	385	217,510	760	1018	1.848	4.966	74	1606	0.285	1784	1.204	722	2407	0.254
	Mean	216	206,594	474	665	1.437	3.222	59	1033	0.165	1228	0.864	408	1263	0.184
	Alloying element content range (wt%): C 0.02–0.50; Si 0.02–0.55; Mn 0.03–1.50; P 0.01–0.05; S 0.01–0.05; Cr 0.02–0.19; Mo 0.00–0.01; Ni 0.01–0.13; Cu 0.01–0.21; Al 0.04–0.07; N 0.00–0.01														
LA	Min	23	187,500	330	540	1.038	2.700	3	717	0.007	926	0.026	322	894	0.067
	Max	357	221,000	1927	2016	1.855	9.739	76	2586	0.236	2230	1.450	1341	3328	0.225
	Mean	225	201,658	857	987	1.184	4.910	56	1298	0.084	1522	0.847	647	1419	0.125
	Alloying element content range (wt%): C 0.04–1.02; Si 0.06–0.68; Mn 0.01–1.43; P 0.01–0.04; S 0.00–0.06; Cr 0.03–1.89; Mo 0.01–1.13; Ni 0.02–1.90; Cu 0.01–0.57; Al 0.00–0.17; Co 0.00–0.21; Ti 0.00–0.04; V 0.06–0.24; Nb 0.00–0.03														
HA	Min	35	172,625	177	516	1.231	2.457	46	349	0.062	1360	1.010	197	987	0.093
	Max	337	210,000	795	1158	3.670	5.521	83	1416	0.362	2407	1.715	882	8384	0.469
	Mean	168	205,811	331	685	2.428	3.342	71	791	0.157	1837	1.402	377	2567	0.305
	Alloying element content range (wt%): C 0.02–0.25; Si 0.27–1.50; Mn 0.41–2.00; P 0.02–0.04; S 0.00–0.02; Cr 11.40–25.00; Mo 0.02–2.62; Ni 0.21–24.65; Cu 0.06–0.31; Al 0.00–0.26; Co 0.11–0.22; Ti 0.00–2.50; W 0.00–0.99; V 0.00–0.28; N 0.05–0.25														

Note: Shaded cells mean that information was not available for all datasets.

**Table 3:** Training parameters of the ANN models.

Parameter	Value
Training algorithm	Levenberg–Marquadt (with early stopping)
Normalization	minmax in the range from −1 to 1
Number of hidden layers	1
Number of neurons per hidden layer	1 to *H* by step 1 UA: 2 data points *H* = 13; 5 data points *H* = 33 LA: 2 data points *H* = 18; 5 data points *H* = 46 HA: 2 data points *H* = 6; 5 data points *H* = 16
Control random number generation	rng(‘default’), Mersenne Twister generator with seed 0
Number of trainings per architecture	10
Training goal	0.0001*σ*_target_^2^
Epochs	1000 (if *MSE* not met)
Cost function	*MSE*
Transfer function	tansig; purelin
Number of *k*-folds	10
Division of data into *k*-fold	random permutation
*σ*_target_^2^ variance of the target variable	
*MSE* mean square error	
Tansig: hyperbolic tangent sigmoid transfer function	
Purelin: linear transfer function	

**Table 4:** Artificial neural networks chosen for estimation of cyclic stress–strain behavior of unalloyed, low-alloy, and high-alloy steels.

Steel Group	Data Points Used for Estimation	*H*	*RMSE*_mean,test_	*r*_test_
UA	2	12	57	0.943
	5	17	75	0.875
LA	2	13	82	0.953
	5	19	93	0.932
HA	2	5	151	0.922
	5	10	137	0.920

**Table 5:** Summary of models’ performance indicators.

Steel Group	Method/Approach	*MAPE*	*RMSE*
UA	Zhang 1 [2]	26.13	182.78
	Zhang 1 [2]	34.08	192.79
	Lopez 1 [4]	12.92	68.28
	Lopez 1 [4]	11.82	59.02
	Li [5]	8.17	44.07
	ANN-param [22]	6.83	39.81
	ANN-CSSC-2	**6.19**	**38.18**
	ANN-CSSC-5	9.47	57.61
LA	Zhang 1 [2]	37.96	270.33
	Zhang 1 [2]	19.79	157.46
	Lopez 1 [4]	6.38	56.28
	Lopez 1 [4]	11.54	78.23
	Li [5]	**5.88**	54.06
	ANN-param [22]	9.06	61.70
	ANN-CSSC-2	6.48	**48.92**
	ANN-CSSC-5	7.70	59.82
HA	Zhang 1 [2]	82.64	332.49
	Zhang 1 [2]	28.05	175.82
	Lopez 1 [4]	20.11	173.56
	Lopez 1 [4]	22.19	183.31
	Li [5]	20.91	129.90
	ANN-param [22]	14.96	136.43
	ANN-CSSC-2	9.62	68.52
	ANN-CSSC-5	**8.98**	**68.05**

Note: Bold values designate lowest values of *MAPE* and *RMSE* for each steel subgroup.

## Data Availability

The data used in this study are available in [9,22].
